# History of Medicine in the West

**Published:** 1852-02

**Authors:** 


### HISTORY OF MEDICINE IN THE WEST.

A highly interesting contribution to the history of the medical profession in the West has just been made by Professor Drake, in an address delivered before the physicians of Cincinnati on the occasion of founding a public library. We hope to be able in our next number to make some extracts from the address. It is the beginning, we trust, of a systematic effort to collect and record the scattered fragments of our professional history. Soon it will be too late to write the history of the pioneers of medicine in the West.

